# Facebook Interaction as a Potential Marker of Cognitive Decline

**DOI:** 10.1002/alz.094856

**Published:** 2025-01-09

**Authors:** Esraa M Qansuwa, Abdelhamid Mohamed, Aliaa Elbekery, Passent El‐Kafrawy, Seif Eldawlatly, Mohamed Salama

**Affiliations:** ^1^ Global Health& Human Ecology Institute, The American University in Cairo, New Cairo, Cairo Egypt; ^2^ menofia univerty, Cairo, Cairo Egypt; ^3^ American Unicersity in Cairo, New Cairo, Cairo Egypt; ^4^ Kafr‐el sheikh University, Cairo, Egypt, Kafr‐el sheikh Egypt; ^5^ The American University in Cairo, Cairo, Cairo Egypt; ^6^ The American University in Cairo, Cairo Egypt

## Abstract

**Background:**

The growth of social media and the continuous improvement of machine‐learning algorithms suggest that social media‐based screening methods for mental diseases will become increasingly feasible with high accuracy in the next few years. Additionally, Artificial Intelligence, particularly predictive machine learning (ML) Models, has been established as one of the more powerful approaches to building reliable models that might be useful as an early predictor for Mental disorders. Specifically, one of the current challenges in brain disorders is identifying patients with Mild Cognitive Impairment (MCI) that might be converted to Alzheimer’s (AD) or other types of dementia. Cognitive decline in older adults is associated with decreased social interaction and behavioral changes features.

**Method:**

Using vast quantities of historical Facebook data from participants aged 50 and above who conducted the MoCA test as a Cognitive Assessment Tool, checking the changes in their written language mistakes and a range psychological features derived from Natural Language Processing (NLP) algorithms, LIWC 2022 dataset, and emotion dataset, using supervised Classification ML tools, such as support Vector Machine Model (SVM) and XGB Model to predict MCI using among (n = 225) participants. We consider formulating Language mistakes and social interactions. Then, we will perform a longitudinal and cross‐sectional examination of the data to examine the changes in a range of features extracted from Facebook subjects’ data over time.

**Result:**

SVM and XGB classification machine learning models show significant promising predictive results, differentiating between patients and normal subjects. SVM Recall is 66%. XGB Recall is 67.5%. Therefore, using our suggested pipeline, which shows promising primary results in building a predictive model, can detect MCI in the early stages.

**Conclusion:**

Facebook data could allow us to investigate the relationship between objective daily activity patterns and objective psychological digital markers of cognitive decline. This would be a promising project that provides an ML model for Facebook activity features among subjects that can be correlated to cognitive changes. Additionally, it matches common diagnostic practices with knowledge acquired from Facebook. We seek to scale this project up globally and build decision support for a diagnostic framework for practitioners that can be used independently.

